# Dataset on the questionnaire-based survey of the readiness of government accountants to implement international public sector accounting standards (IPSAS)

**DOI:** 10.1016/j.dib.2025.111302

**Published:** 2025-01-18

**Authors:** Moawiah Awad Alghizzawi

**Affiliations:** Department of Managerial & Financial Sciences, Al Zahra College for Woman, Muscat, Oman

**Keywords:** Questionnaire survey, IPSAS, Human resource, Information technology, Public sector

## Abstract

This article presents a data set concerning the effect of human resource and information technology on the readiness of government accountants to implement IPSAS. The data was collected by a face-to-face survey with a questionnaire using five point Likert scale. The survey sample involved 331 government accountants in Jordan. The questionnaire consists of three sections. Section A addresses the demographic attributes of the respondents. Section B contains 2 parts; part (1) measures human resource, part (2) measures information technology. Section C measures the readiness of government accountants to implement IPSAS. Each section contains seven to eight statements with a 1-5 Likert scale in order to assess respondents’ level of agreement or disagreement. The detailed data set is accompanied by the original questionnaire in English and Arabic. The data set is providing a valuable tool to understand the contributions of human resource and information technology in achieving the readiness to implement IPSAS, develop hypotheses and design a questionnaire survey.

Specifications Table

**Table:** 

Subject	Accounting.
Specific subject area	International Public Sector Accounting Standards.
Type of data	Table.
Data collection	The data derived from the previous research served to develop the structured questionnaire. The questionnaire sample involved 331 government accountants working in the Jordanian Ministry of Finance (JMOF). Simple random sampling strategy was selected given the sameness of the characteristics of the targeted respondents and the need to acquire a representative sample for the population of interest. The data were hand-collected using a face-to-face questionnaire available in two languages – English and Arabic. following sections were the matters of interest of the data: - demographic attributes of the respondents - human resource - information technology - readiness of government accountants to implement IPSAS
Data source location	Institution: Al Zahra College for Woman City/Town/Region: Muscat Country: Sultanate of Oman
Data accessibility	Repository name: Mendeley Data Data identification number: 10.17632/725x4sgnsw.3 Direct URL to data: https://data.mendeley.com/datasets/725x4sgnsw/3

## 1 Value of the Data

• The data can be used for further future research to interpret the relationship between human resources and the information technology system and its impact on the readiness of government accountants to implement IPSAS. Therefore, it contributes to the development of new explanatory models and theories related to this relationship.
• The data is providing a valuable tool to understand the contributions of human resource and information technology in achieving the readiness to implement IPSAS, develop hypotheses and design a questionnaire survey.
• The data can be useful in implementing IPSAS. Therefore, improving the financial reports produced by the public sectors. This in turn enhancing confidence by investors and other official bodies, and thus the overall performance of the public sectors.
• The data enables decision makers to better guide future policies and strategies to improve the readiness to implement IPSAS, ascertaining that government accountants adhere in accordance with IPSAS implementation. This can be achieved by enabling policy makers to target specific gaps in compliance and readiness, identifying the needed support (e.g., training, system updates, resource allocation) and therefore, providing a clear guideline for government accountant.

## 2 Background

The readiness of government accountants, which implies their desire to change the way government bodies keep their books, is a key determinant of optimal IPSAS implementation [1]. These accountants must be provided with the necessary resources to accommodate the new system [2]. For example, human resources (HR) must be equipped with optimal information technology (IT) systems [3] and effectively managed and educated [4] to ensure a seamless implementation process and accommodate the new change towards IPSAS. Although the government has developed a robust plan to implement IPSAS, Jordanian research on these variables remains lacking [5]. Jordan is the first country in the middle east started to implement IPSAS. In 2015, the government of Jordan successfully adopted cash accounting based on IPSAS towards the full implementation of IPSAS by 2021. However, the full IPSAS implementation is not achieved yet according to the financial statements reports establishing by Jordanian Ministry of Finance.

## 3 Data Description

A Microsoft Excel Worksheet was attached with this article as supplementary material. The data providing respondents agreements or disagreements in regards to the statements of questionnaire. All the statements have 331 entries of the respondents. A 1–5 Likert scale (1. Strongly Disagree, 2. Disagree, 3. Neutral, 4, Agree & 5. Strongly agree) was employed to enable the respondents to easily complete the questionnaire survey while increasing the response rate and quality [6]. The detailed data set is accompanied by the original questionnaire in English and Arabic.

For the purpose of conducting a pilot study, it is recommended to use a sample size that is 10% of the intended sample size for the main study (De Vaus, 2013). Hence, preliminary research undertaken to assess the accuracy and consistency of the tool. A preliminary investigation was carried out for this study, the investigation involved a group of 55 government accountants from the Income Sales & Tax Department and Financial Directorates. The pilot study obtained 55 questionnaires that were circulated. Then 41 questionnaires were return from the total; but also, had to exclude five surveys that were deemed invalid for analysis. Finally, 36 questionnaires were deemed usable based on the efforts mentioned above. Subsequently, equation modelling was employed to evaluate the dependability of the items by using the outcomes of Cronbach's alpha coefficient (α) via SPSS software. Based on the Cronbach's alpha coefficient results, the values for all human resource, information technology, and readiness of government accountants to implement IPSAS items are 0.915, 0.912, and 0.916, respectively. Items with values of 0.70 and above demonstrated adequate reliability (Nunnally & Bernstein, 1994).

## 4 Experimental Design, Materials and Methods

The data derived from the previous research served to develop the structured questionnaire. Eight “human resource” items were derived from [7,8], while eight “information technology” items were derived from [9,10]. Meanwhile, seven “readiness of government accountants to implement IPSAS” items were developed following [11,12].

As the respondents hailed from Jordan, the instrument was translated into Arabic language. The developed questionnaire items must be reviewed by experts to achieve the data validity. As such, 10 governmental accounting experts were chosen to validate the questionnaire. Expert feedback on the data variables corroborated the measurements derived from current works. Consequently, the instrument was prepared following the existing literature. Some experts suggested improvements on the items (sentence structure) to avoid ambiguities when translating into the Arabic language.

Content validity index (CVI) was used in order to quantify multi-item scales’ content validity [13]. Content validity assessment depended on measurement scale representativeness following a five-point rating scale [14] proposed retaining items exceeding 0.80 and omitting those under 0.80. Table 1 displays CVI of the data:

**Table1:** Content validity index (CVI).

Items	Expert 1	Expert 2	Expert 3	Expert 4	Expert 5	Expert 6	Expert 7	Expert 8	Expert 9	Expert 10	CVI	
HR1	4	4	3	3	4	4	3	4	3	3	10/10	1.0
HR2	4	4	4	3	4	4	3	4	1	3	9/10	0.9
HR3	3	3	2	4	4	4	3	3	3	4	9/10	0.9
HR4	4	4	4	3	4	4	3	1	4	4	9/10	0.9
HR5	3	4	4	2	4	4	3	1	4	3	8/10	0.8
HR6	3	4	4	3	4	4	3	4	2	3	9/10	0.9
HR7	4	3	4	3	4	2	3	2	4	4	8/10	0.8
HR8	3	4	3	4	4	3	4	4	3	4	10/10	1.0
IT1	4	2	1	4	3	4	3	4	3	4	8/10	0.8
IT2	4	4	4	3	3	4	3	4	3	3	10/10	1.0
IT3	3	4	4	4	2	4	4	3	4	3	9/10	0.9
IT4	2	3	2	4	3	4	3	3	3	3	8/10	0.8
IT5	2	4	4	4	4	4	4	1	3	3	8/10	0.8
IT6	4	4	4	4	4	4	4	3	4	3	10/10	1.0
IT7	4	4	4	4	4	4	4	3	3	4	10/10	1.0
IT8	4	2	3	4	3	4	4	2	3	4	8/10	0.8
RGA1	4	4	3	3	4	4	4	4	4	3	10/10	1.0
RGA2	4	4	3	4	4	1	4	3	3	2	8/10	0.8
RGA3	4	3	3	2	4	4	4	3	4	4	9/10	0.9
RGA4	4	1	1	3	4	4	4	4	3	4	8/10	0.8
RGA5	4	2	2	4	4	4	4	4	3	4	8/10	0.8
RGA6	4	3	4	3	4	4	4	4	2	4	9/10	0.9
RGA7	4	4	4	2	4	4	4	2	4	3	8/10	0.8

The population of the data set represent the Jordanian public sector. Specifically, the Jordanian Ministry of Finance (JMOF), which consists of six departments with the ministry itself, the targeted population of the data, consists of all government accountants working in the JMOF with its departments as indicating in Table 2. The majority of the respondents of the data are dominated by male. This may be as the result of population structure (Government Accountants) of Jordanian JMOF where male constitutes 71.3 % and female 28.7 %.

**Table 2:** Population of the data.

#	Ministry of Finance		General Budget Department		Customs Department		Land & Surveys Department		General Supplies Department		Income Tax & Sales Department	
Government Accountants	M	F	M	F	M	F	M	F	M	F	M	F
	442	204	10	8	190	60	246	123	4	2	498	161
Total	646	18	250	369	6	659						
Total in all departments	1948 Government Accountants											

The probability sampling strategies include specific techniques that assist to sample the population of interest where all units of the population have similar probability of being selected. Simple random sampling strategy was selected given the sameness of the characteristics of the targeted respondents and the need to acquire a representative sample for the population of interest. Following [15], the target sample size of this data is 321 participants (margin of error = 5 %). The determined sample of this data was appropriate, going by the [16] rule of thumb. Table 3 shows different sample sizes at a 95 % level of confidence with different margins of error:

**Table 3:** Distribution of government accountants.

Department	Population		Sample	
	Number of Financial Employees	Percentage	Number of the Sample	Percentage
Ministry of Finance	646	33%	106	33%
General Budget Department	18	1%	3	1%
Customs Department	250	13%	42	13%
Land & Surveys Department	369	19%	61	19%
General Supplies Department	6	1%	3	1%
Income Tax & Sales Department	659	33%	106	33%
Total	N = 1948	100%	S = 321	100%

A face-to-face technique was used; respondents were assured that the given information will be used only for academic purpose. The approximate time to complete the survey was 10 to 15 min. The entire data collection period was about eight weeks. A total of 375 questionnaires were distributed to government accountants. Each questionnaire had been verbally scanned to clean the data from any missing responses. A total of 331 questionnaires were collected and used for subsequent analysis, giving a response rate of 88 %. This rate was due to the fact that the questionnaires were personally distributed by hand. Table 4 illustrates the response rate:

**Table 4:** Response rate.

Number of questionnaires distributed	375
Incomplete / Not returned questionnaire	44
Useable questionnaires	331
Questionnaires response rate	88%

Respondents were approached individually to verify that their responses were independent and unbiased. The questionnaire was handed out on paper to ensure accessibility, especially for respondents who might face challenges with digital platforms. Following that, the responses from paper questionnaires were manually entered into Excel. To reduce errors, two independent researchers cross-checked the entries. Furthermore, random samples were verified for consistency, and any inconsistencies were resolved by discussion.

The Demographical Attributes of the data included gender, work experience, level of education, educational background.

The first demographic item pertained to the gender of the respondents. In accordance with the statistics presented in Table 5, the data shows that 61.9% of the respondents were male, while the remaining 38.1% were female. The second item inquired about the work experience of the respondents. Table 5illustrates that the largest proportion of respondents had between 11 and 20 years of experience (44.4%), followed by under 10 years (30.2%), 21–30 years (35.1.3%), and the smallest group consisted of respondents with more than 31 years of experience (0.3%). In relation to the item three that represent the educational of the participants, they were requested to indicate their degree of schooling. As depicted in Table 5, the predominant educational attainment among respondents was a bachelor's degree (61.9%). This was followed by diploma's degrees (15.1%), master's degrees (14.8%) and high school (5.4%). The lowest educational level reported by respondents was a doctorate, which constituted 2.7% of the sample. Lastly, participants were requested to indicate their Background. As depicted in Table 5, the majority of participants are accounting and finance (69.5%), Followed by others such economic (13.3%), business administration (10%), public administration (5.4%), and the lowest percentage belonged to law & political science (1.8%).

**Table 5:** Demographical attributes of the respondents.

#	Profile	Frequency	Percentage
Gender	1. Male	205	61.9
	2. Female	126	38.1

Experience	1. Under 10 years	100	30.2
	2. 21-20 years	147	44.4
	3. 21-30 year	83	25.1
	4. 31 and above	1	0.3

Education	1. High School	18	5.4
	2. Diploma	50	15.1
	3. Bachelor	205	61.9
	4. Master	49	14.8
	5. Doctorate	9	2.7

Background	1. Accounting and finance	230	69.5
	2. Law and political science	6	1.8
	3. Business administration	33	10
	4. Public administration	18	5.4
	5. Others	44	13.3

Data with non-normal distribution were either skewed to the left or right based on the kurtosis variables [17], which generated inconsistent outcomes concerning the variable correlations and their significance. Univariate normality was evaluated based on skewness and kurtosis values. Both skewness and kurtosis values should reflect ±2 and ±7, respectively [18]. Table 6 presents the skewness and kurtosis values, which ranged from -1.029 to -0.227 and from -0.278 to 1.949, respectively. Overall, the survey data revealed normal distribution:

**Table 6:** Assessment of normality of all items.

Construct	Item	Skewness	Std. Error of Skewness	Kurtosis	Std. Error of Kurtosis
Human Resource (HR)	HR1	-0.373	0.134	0.06	0.267
	HR2	-0.454	0.134	0.005	0.267
	HR3	-0.319	0.134	-0.278	0.267
	HR4	-0.354	0.134	0.266	0.267
	HR5	-0.778	0.134	1.087	0.267
	HR6	-0.317	0.134	0.451	0.267
	HR7	-0.654	0.134	0.976	0.267
	HR8	-0.366	0.134	0.56	0.267
Information Technology (IT)	IT1	-0.685	0.134	0.704	0.267
	IT2	-0.794	0.134	1.339	0.267
	IT3	-1.029	0.134	1.949	0.267
	IT4	-0.734	0.134	1.05	0.267
	IT5	-0.567	0.134	0.502	0.267
	IT6	-0.75	0.134	1.136	0.267
	IT7	-0.757	0.134	1.136	0.267
	IT8	-0.38	0.134	0.279	0.267
Readiness of Government Accountants for IPSAS implementation (RGA)	RGA1	-0.733	0.134	1.012	0.267
	RGA2	-0.227	0.134	0.175	0.267
	RGA3	-0.491	0.134	0.524	0.267
	RGA4	-0.673	0.134	0.843	0.267
	RGA5	-0.397	0.134	-0.009	0.267
	RGA6	-0.558	0.134	0.623	0.267
	RGA7	-0.366	0.134	-0.119	0.267

Table 7 presents the obtained mean (a measure of central tendency) and standard deviation (dispersion index) of each construct according to the five-point Likert scale:

**Table 7:** Descriptive statistics.

Constructs	Mean	Standard Deviation	Minimum	Maximum
Human Resource	3.570	0.645	1.1	4.9
Information Technology	3.678	0.579	1	5
Readiness of Government Accountants to Implement IPSAS	3.759	0.619	1.7	5

**Fig. 1:**
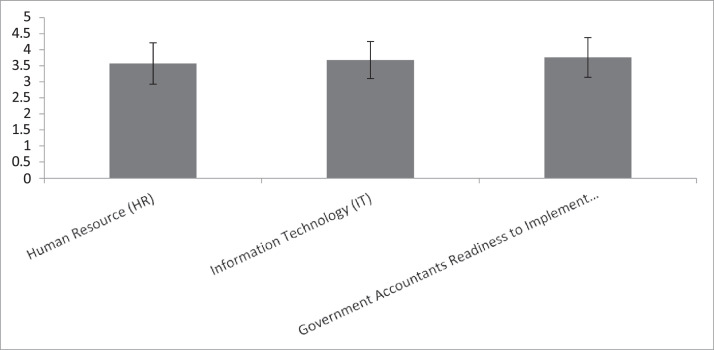
Means and standard variations of all variables.

Fig. 1 provides a good illustration for the mean of all variables together with their standard deviations:

The mean was utilised as a metric for central tendency, revealing that the average values of Human Resource (HR), Information Technology (IT) and Readiness of Government Accountants to Implement IPSAS (RAG) exceeded the middle threshold of 3 on a 5-point Likert scale. The phenomenon demonstrated that the perceptions of the respondents towards these variables were higher than the average. The standard deviation was utilised as a measure of dispersion to highlight the extent to which individuals within each variable deviate from the variable mean. Out of all the variables that were examined, the individual value of Human Resource (HR) had the highest deviation from its average value (standard deviation = 0.645). Conversely, Information Technology (IT) had the smallest variation from the average, with a standard deviation of 0.579.

## Limitations

A noteworthy limitation of the data is the observable bias towards male participants within the sample population. The presence of gender bias could potentially influence the results and restrict their applicability to the wider populations. Several factors, including the method of data acquisition, sample selection criteria, and societal influences on participation rates, may have contributed to this bias. Beyond that, the results may continue to offer valuable insights into trends, enable the identification of recurring patterns, or inspire the development of hypotheses for future investigations. Moreover, endeavors to acknowledge and alleviate bias in subsequent investigations may result in research findings that are more comprehensive and timely. Therefore, despite recognizing the limitation, the data continue to be valuable and pertinent in furthering understanding within the discipline.

## Ethics Statement

To ensure the preservation of participant confidentiality, every piece of data gathered throughout the research was anonymized. Individual identifiers were omitted, and any potentially identifying data was substituted with distinct codes. By following this procedure, the privacy and confidentiality of every respondent were guaranteed, in adherence to ethical research principles. Therefore, the Research Ethics Committee of the Al Zahra College for Woman exempted this study from ethical approval.

Due to constraints outlined by the Jordanian Ministry of Finance to obtain a documented informed consent; all participants granted a verbal informed consent. However, all participants were informed about the purpose of the study, their voluntary participation, and the confidentiality & anonymity of their responses. Participants were given the opportunity to ask questions and clarify any concerns they may have had regarding their participation.

## CRediT authorship contribution statement

**Moawiah Awad Alghizzawi:** Conceptualization, Methodology, Data curation, Software, Writing – original draft.

## Data Availability

Mendeley DataDataset: Supplementary Data: ‎readiness of government accountants to implement IPSAS (Original data).

## References

[1] Zakiah S., Isa C.,R., Haslida H. (2013). Accrual accounting: change and managing change. IPN J. Res. Pract. Public Sect. Account. Manag..

[2] IFAC (2013).

[3] OECD/IFAC (2017).

[4] Irvine H. (2011). From go to woe. How a not-for-profit managed the change to accrual accounting. Account. Audit. Account..

[5] Wiggins J., Biggs D., Al-Bokairat O. (2016). AECOM International Development Europe SL.

[6] Babakus E., Mangold W.G. (1992). Adapting the SERVQUAL scale to hospital services: an empirical investigation. Health Services Research,.

[7] Shaw JD., Gupta N., Delery JE. (2005). Alternative conceptualizations of the relationship between voluntary turnover and organizational performance. Acad. Manag. J..

[8] Holt D.T., Armenakis A.A., Feild H.S., Harris S.G. (2007). Readiness for organizational change: the systematic development of a scale. J. Appl. Behav. Sci..

[9] Karimi J., Gupta Y.P., Somers T.M. (1996). Impact of competitive strategy and information technology maturity on firms’ strategic response to globalization. J. Manag. Inf. Syst..

[10] Zhu K., Kraemer K.L., Gurbaxani V., Xu S.X. (2006). Migration to open-standard interorganizational systems: Network effects, switching costs, and path dependency. MIS Q..

[11] Kwahk K.Y, Lee J.N. (2008). The role of readiness for change in ERP implementation: theoretical bases and empirical validation. Inf. Manag..

[12] Bosmans A., Rik P., Hans B. (2010). The get ready mind-set: how gearing up for later impacts effort allocation now. J. Consum. Res..

[13] Rubio D.M., Berg-Weger M., Tebb S.S., Lee E.S., Rauch S. (2003). Objectifying content validity: conducting a content validity study in social work research. Soc. Work Res..

[14] Davis L.L. (1992). Instrument review: getting the most from a panel of experts. Appl. Nurs. Res..

[15] Krejcie R.V, Morgan D.W. (1970). Determining sample size for research activities. Educ. Psychol. Meas..

[16] Roscoe J.T. (1975).

[17] Brown C.E. (2012).

[18] Ho R. (2006).

